# The *Longissimus* and *Semimembranosus* Muscles Display Marked Differences in Their Gene Expression Profiles in Pig

**DOI:** 10.1371/journal.pone.0096491

**Published:** 2014-05-08

**Authors:** Frederic Herault, Annie Vincent, Olivier Dameron, Pascale Le Roy, Pierre Cherel, Marie Damon

**Affiliations:** 1 INRA, UMR1348, PEGASE, F-35590 Saint-Gilles, France; 2 Agrocampus Ouest, UMR1348, PEGASE, F-35000 Rennes, France; 3 Université de Rennes1, F-35000 Rennes, France; 4 IRISA team Dyliss, F-35000 Rennes, France; 5 iBV-institut de Biologie Valrose, Université Nice-Sophia Antipolis UMR CNRS 7277 Inserm U1091, Parc Valrose, F-06108 Nice, France; University of Bonn, Germany

## Abstract

**Background:**

Meat quality depends on skeletal muscle structure and metabolic properties. While most studies carried on pigs focus on the *Longissimus* muscle (LM) for fresh meat consumption, *Semimembranosus* (SM) is also of interest because of its importance for cooked ham production. Even if both muscles are classified as glycolytic muscles, they exhibit dissimilar myofiber composition and metabolic characteristics. The comparison of LM and SM transcriptome profiles undertaken in this study may thus clarify the biological events underlying their phenotypic differences which might influence several meat quality traits.

**Methodology/Principal Findings:**

Muscular transcriptome analyses were performed using a custom pig muscle microarray: the 15 K Genmascqchip. A total of 3823 genes were differentially expressed between the two muscles (Benjamini-Hochberg adjusted P value ≤0.05), out of which 1690 and 2133 were overrepresented in LM and SM respectively. The microarray data were validated using the expression level of seven differentially expressed genes quantified by real-time RT-PCR. A set of 1047 differentially expressed genes with a muscle fold change ratio above 1.5 was used for functional characterization. Functional annotation emphasized five main clusters associated to transcriptome muscle differences. These five clusters were related to energy metabolism, cell cycle, gene expression, anatomical structure development and signal transduction/immune response.

**Conclusions/Significance:**

This study revealed strong transcriptome differences between LM and SM. These results suggest that skeletal muscle discrepancies might arise essentially from different post-natal myogenic activities.

## Introduction

Pork is one of the most widely eaten meats in the world. Breeding programs aiming at improving pig production efficiency through increased growth rate and lean meat content and decreased fatness have also affected some meat quality traits playing an important role in consumer acceptance of pork like water holding capacity, color, intramuscular fat (IMF) content and tenderness [1]. Meat quality is a complex trait which depends on the interactive effects of pig genotype, environmental conditions, pre-slaughter handling and slaughtering procedure [2]. The skeletal muscle structure and metabolic characteristics which determine cellular and molecular events occurring during muscle to meat transformation are of the utmost importance for meat quality determination. Skeletal muscle is a heterogeneous tissue composed of myofibers, adipose, connective, vascular and nervous tissues. Myofibers differ by their molecular, structural, contractile and metabolic properties according to which they are classified as slow-twitch oxidative (type I), fast-twitch oxido-glycolytic (type IIA) and fast-twitch glycolytic (type IIB). Red or white muscles are also determined according to their fiber type composition. Red muscles are composed of high percentage of slow-twitch oxidative fibers whereas white muscles contain a major proportion of fast-twitch glycolytic fibers [3]. *Longissimus* and *Semimembranosus* - two white skeletal muscles - are consumed in different forms: fresh for LM (loin) or after processing for SM (ham). Both muscles are classified as glycolytic even if slight differences have been described in their myofiber composition (higher proportion of type IIa myofiber and lower proportion of Type IIb myofiber in SM) and metabolic properties (higher oxidative capacity in SM) [4]–[7]. Transcriptome analysis might be useful to identify transcriptional signatures associated with meat quality traits which could thus be selected as biomarkers in selection programs [8]–[12]. However, pig transcriptome studies are mainly focused on LM even if gene expression variability between muscles could affect muscle development, meat quality and hence the choice of meat processing [13].

The aim of this study was to better characterize LM and SM gene expression profiles in order to investigate the biological events underlying their distinct metabolic and contractile properties.

## Results

### Comparison of Gene Expression Profiles between *Longissimus* and *Semimembranosus* Muscles

**Figure 1:**
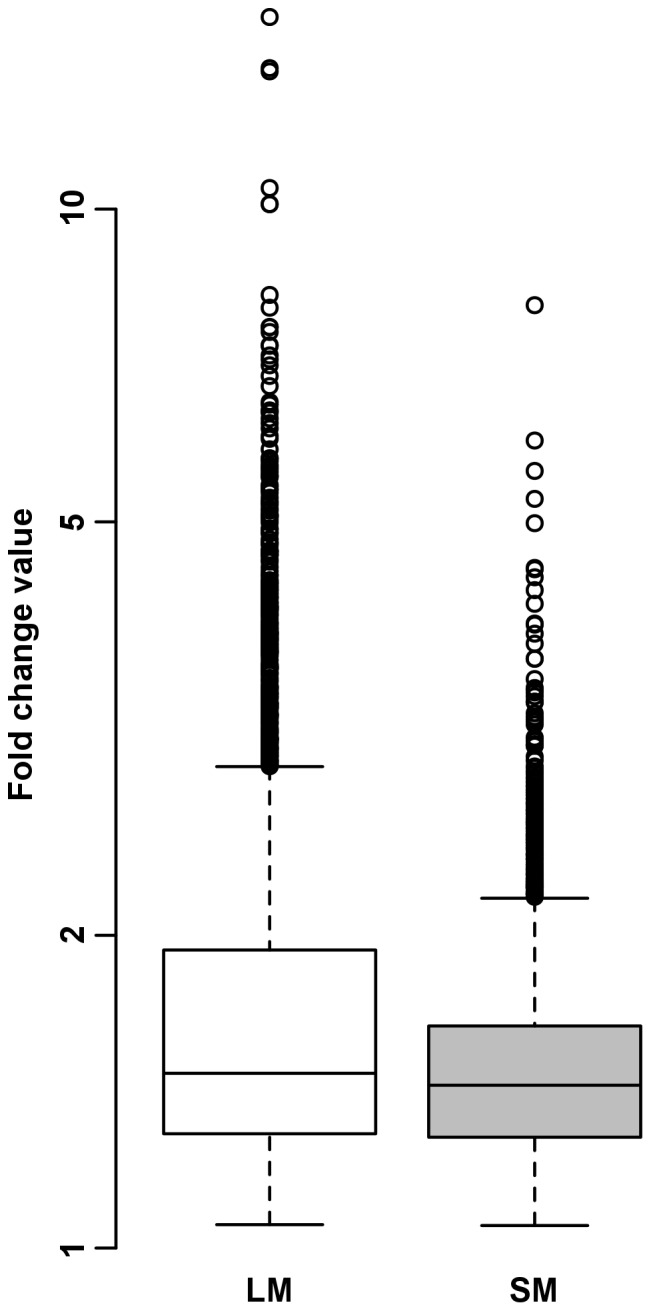
Gene expression ratio between muscles. Muscle fold change ratio is expressed as the expression ratio of *Longissimus* (LM) to *Semimembranosus* (SM) samples when genes are highly expressed in *Longissimus* and as the expression ratio of SM to LM samples when genes are highly expressed in *Semimembranosus*.

Gene expression microarray analysis was conducted on 180 muscle samples (90 LM, 90 SM). Comparison of LM and SM muscle transcriptome was achieved using the “GenmascqChip”, a 15 k pig skeletal muscle microarray. Raw data sets were checked for quality criteria. The 10753 remaining probes were considered significantly expressed in both muscles. We observed a strong muscle effect on gene expression with 5582 (37%) of probes being differentially expressed between LM and SM (adjusted P value ≤0.05). As shown in Figure 1, fold change (FC) ratios varied from 1.1 to 15 and were for the most part quite low with median values <1.5 in the two muscles. These 5582 differentially expressed probes corresponded to 3823 annotated genes, with 1690 and 2133 genes overrepresented in LM (Table S1) and SM (Table S2), respectively. A set of 2402 differentially expressed probes (1603 annotated genes) with a muscle FC ratio above 1.5 was considered as biologically relevant and selected for functional analysis.

The ten most differentially expressed and informative genes [i.e. with at least one associated gene ontology (GO) biological process (BP) term] are shown in Table 1 for LM (5.3≤FC≤15.3) and in Table 2 for SM (3.4≤FC≤8.1). Among the ten genes strongly expressed in the LM, three are involved in gene expression: poly(rC) binding protein 2 (*PCBP2*), microphthalmia-associated transcription factor (*MITF*) and zinc finger and BTB domain-containing protein 16 (*ZBTB16*). Three other genes are involved in metabolism: ATP synthase, H+ transporting, mitochondrial Fo complex, subunit E (*ATP5I*), ADAM metallopeptidase with thrombospondin type 1 motif, 8 (*ADAMTS8*) and kinase D-interacting substrate, 220 kDa (*KIDINS220*). *ADAMTS8* and *ZBTB16* are also related to negative regulation of cell proliferation. Two genes are involved in muscle development: interferon-related developmental regulator 1 (*IFRD1*) and ryanodine receptor 1 (*RYR1*) which is also involved in muscle contraction and calcium ion transport. Last, one gene is related to cell-cell signaling: nudix (nucleoside diphosphate linked moiety X)-type motif 3 (*NUDT3*) and one gene in DNA replication and DNA repair: REV3-like, polymerase (DNA directed), zeta, catalytic subunit (*REV3L*). For the SM, four genes are involved in gene expression: spleen focus forming virus proviral integration oncogene (*SPI1*), nuclear receptor subfamily 2, group C, member 2 (*NR2C2*), histone cluster 1, H2ab (*HIST1H2AB*) and tenascin C (*TNC*) which is also related to positive regulation of cell proliferation. Two genes are involved in muscle contraction: protein phosphatase 1, regulatory subunit 12B (*PPP1R12B*) and myosin, heavy chain 11 (*MYH11*). Last, one gene is involved in wounding and inflammation (acid phosphatase 5, tartrate resistant, *ACP5*), one gene in water transport (aquaporin 4, *AQP4*), one gene in cellular component movement (kinesin family member C2, *KIFC2*) and one gene in cell adhesion (secreted phosphoprotein 1, *SPP1*).

**Table 1:** Genes overexpressed in *Longissimus* muscle (n = 10).

Symbol^1^	Cluster^2^	FC^3^	P-value^4^	Associated GO BP terms^5^
**ADAMTS8**	4	15.3	<1E^−12^	GO:0008285∼Negative regulation of cell proliferation
				GO:0006508∼Proteolysis
**RYR1**	4	13.6	<1E^−12^	GO:0006816∼Calcium ion transport
				GO:0048741∼Skeletal muscle fiber development
				GO:0006936∼Muscle contraction
**REV3L**	2	8.3	<1E^−12^	GO:0006261∼DNA-dependent DNA replication
				GO:0006281∼DNA repair
**IFRD1**	4	6.4	<1E^−12^	GO:0007518∼Myoblast cell fate determination
				GO:0042692∼Muscle cell differentiation
				GO:0007527∼Adult somatic muscle development
**PCBP2**	3	6.4	<1E^−12^	GO:0008380∼RNA splicing
				GO:0010467∼Gene expression
				GO:0016071∼mRNA metabolic process
**NUDT3**	5	6.3	<1E^−12^	GO:0007267∼Cell-cell signaling
**ATP5I**	1	5.8	<1E^−12^	GO:0022904∼Respiratory electron transport chain
				GO:0006200∼ATP catabolic process
				GO:0042776∼Mitochondrial ATP synthesis coupled proton transport
**KIDINS220**	2	5,4	<1E^−12^	GO:0000186∼Activation of MAPKK activity
				GO:0048011∼Nerve growth factor receptor signaling pathway
**MITF**	3	5,3	<1E^−12^	GO:0007275∼Multicellular organismal development
				GO:0045893∼Positive regulation of transcription, DNA-dependent
**ZBTB16**	4	5,3	<1E^−12^	GO:0006915∼Apoptotic process
				GO:0008285∼Negative regulation of cell proliferation
				GO:0045893∼Positive regulation of transcription, DNA-dependent
				GO:0045892∼Negative regulation of transcription, DNA-dependent

1 Only genes with at least one associated GO BP term are presented in the table.

2 Differentially expressed genes were clustered using GO BP terms semantic similarity between genes as distance, to group functionally similar genes together.

3 Fold Change is expressed as the expression ratio of *Longissimus* to *Semimembranosus* samples.

4 Benjamini and Hochberg adjusted P value.

5 Unique identifier and gene ontology term in the GO database (http://www.geneontology.org/).

**Table 2:** Genes overexpressed in *Semimembranosus* muscle (n = 10).

Symbol^1^	Cluster^2^	FC^3^	P-value^4^	Associated GO BP terms^5^
**SPI1**	3	8.1	<1E^−12^	GO:0045893∼Positive regulation of transcription, DNA-dependent
				GO:0045892∼Negative regulation of transcription, DNA-dependent
				GO:0045814∼Negative regulation of gene expression, epigenetic
**NR2C2**	4	6	<1E^−12^	GO:0006355∼Regulation of transcription. DNA-dependent
				GO:0030154∼Cell differentiation
				GO:0010467∼Gene expression
**PPP1R12B**	4	5.6	<1E^−12^	GO:0006937∼Regulation of muscle contraction
				GO:0007165∼Signal transduction
**TNC**	4	5.3	<1E^−12^	GO:0008284∼Positive regulation of cell proliferation
				GO:0007528∼Neuromuscular junction development
				GO:0010628∼Positive regulation of gene expression
**SPP1**	4	4.5	<1E^−12^	GO:0007155∼Cell adhesion
				GO:0001649∼Osteoblast differentiation
				GO:0001503∼Ossification
**HIST1H2AB**	4	4.3	<1E^−12^	GO:0006334∼Nucleosome assembly
**AQP4**	4	3.9	<1E^−12^	GO:0006810∼Transport
				GO:0006833∼Water transport
				GO:0050891∼Multicellular organismal water homeostasis
**ACP5**	1	3.7	<1E^−12^	GO:0060349∼Bone morphogenesis
				GO:0050728∼Negative regulation of inflammatory response
**KIFC2**	4	3.5	<1E^−12^	GO:0007018∼Microtubule-based movement
**MYH11**	4	3,4	<1E^−12^	GO:0030241∼Skeletal muscle myosin thick filament assembly
				GO:0048251∼Elastic fiber assembly
				GO:0006936∼Muscle contraction

1 Only genes with at least one associated GO BP term are presented in the table.

2 Differentially expressed genes were clustered using GO BP terms semantic similarity between genes as distance, to group functionally similar genes together.

3 Fold Change is expressed as the expression ratio of *Semimembranosus* to *Longissimus* samples.

4 Benjamini and Hochberg adjusted P value.

5 Unique identifier and gene ontology term in the GO database (http://www.geneontology.org/).

### Quantitative RT-PCR Validation of Microarray Analysis

**Figure 2:**
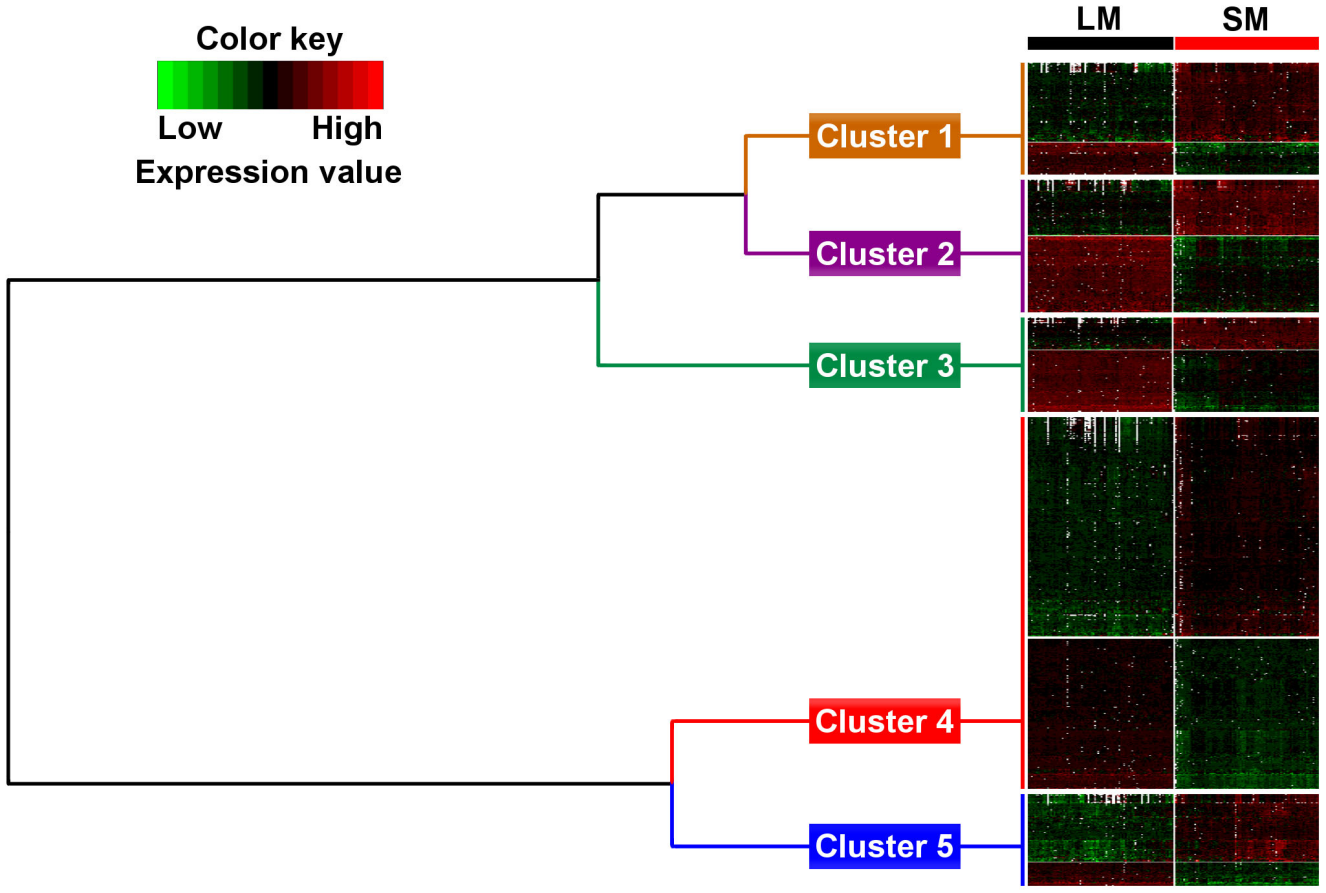
Validation of seven microarray differentially expressed genes between Longissimus (LM) and Semimembranosus (SM) muscles by quantitative RT-PCR. mRNA level is expressed using arbitrary units. Quantitative RT-PCR expression levels (LM = 8, SM = 8) were normalized to the expression of beta 2 microglobulin (*B2M*), TATAbox binding protein (*TBP*) and *18S* using geNorm algorithm. Microarray adjusted means for LM and SM (LM = 90; SM = 90) were calculated using least square means for the muscle effect. Data are expressed as means±s.d. Statistical significances are reported below the plot as Benjamini and Hochberg adjusted P-value for microarray data and as Student t-test P value for q RT-PCR. Fold change ratio is expressed as the expression ratio of LM to SM when genes are overrepresented in LM and as the expression ratio of SM to LM when genes are overrepresented in SM.

Seven target genes, including four genes overrepresented in LM (*ADAMTS8*, *ALDOA*, *CPT1B* and *RYR1*) and 3 genes overrepresented in SM (*CEBPA*, *DGAT2* and *TGFB1*) were analyzed by real time RT-qPCR. These genes were selected to represent the variation of FC ratio observed across the set of 1603 differentially expressed genes with a muscle FC ratio above 1.5. As shown in Figure 2, the comparison of FC ratios between microarray and RT-qPCR technologies provided similar FC direction between these two methods. However, FC values were much less consistent between methodologies for gene overexpressed in LM than in SM samples.

### Functional Analysis

**Figure 3:**
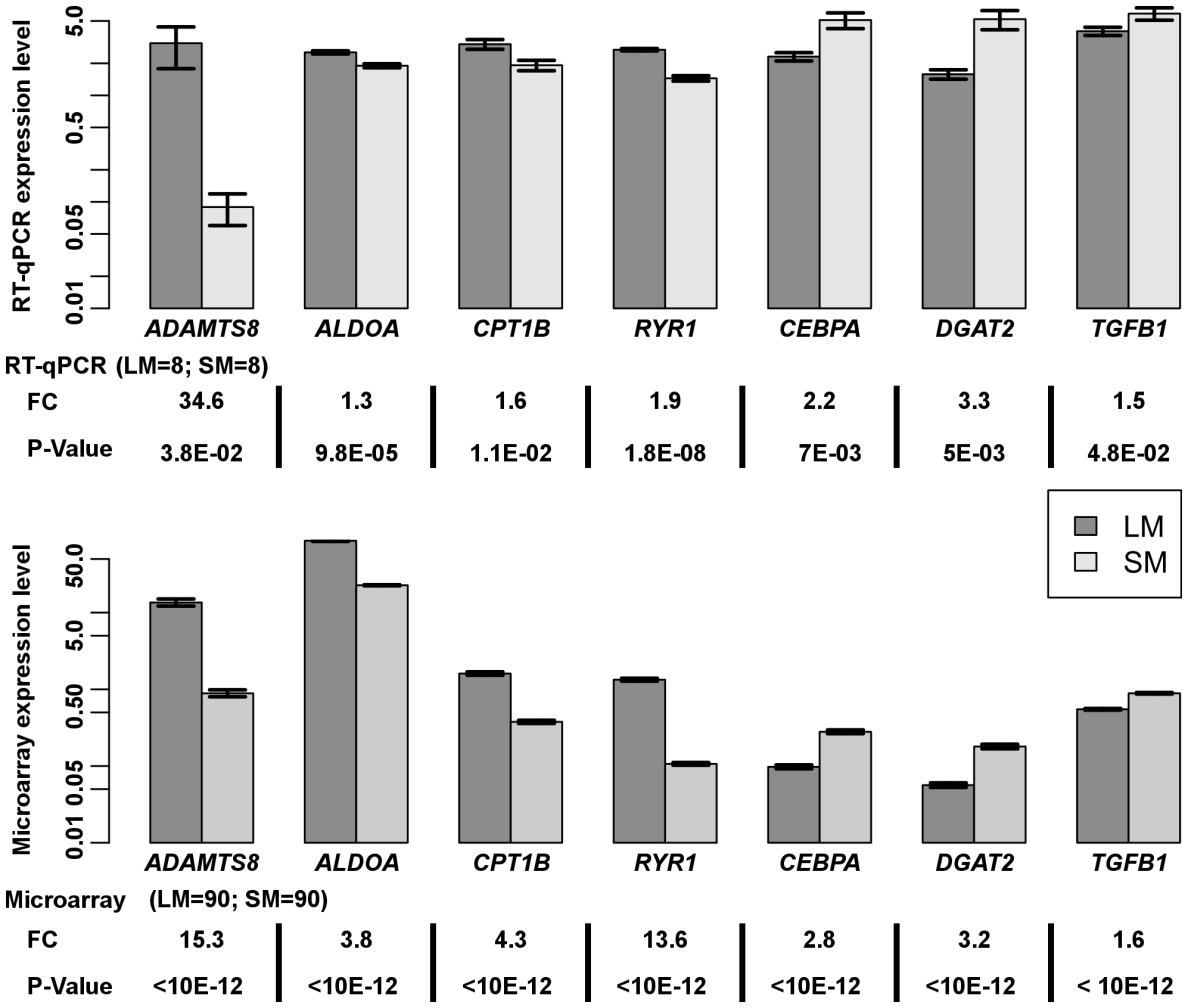
Hierarchical clustering of differentially expressed genes according to their GO BP terms semantic similarity. Annotated differentially expressed genes with a muscle fold change above 1.5 were clustered based on their functional annotation (GO BP) semantic similarity. Hierarchical clustering was performed using “1-semantic similarity” as distance between two genes (similar genes have a distance close to zero) to identify clusters of genes sharing BP terms. Five clusters were identified. Cluster 1 comprised 98 genes highly expressed in SM and 44 in LM. Cluster 2 included 73 highly expressed genes in SM and 102 in LM. Cluster 3 contained 43 highly expressed genes in SM and 84 in LM. Cluster 4 comprised 288 genes overexpressed in SM and 192 in LM. Cluster 5 involved 90 overexpressed genes in SM and 33 overexpressed genes in LM.

To identify the biological events to which differentially expressed genes product contributes, we used GO BP annotation. A set of 2402 differentially expressed probes with a muscle FC ratio above 1.5 was selected for functional analysis. They corresponded to 1603 human orthologous genes. Among them, 1047 were associated with at least one GO BP term and were clustered according to their semantic similarities using these terms (Figure 3). Cluster compositions are shown in Table S3.

Five clusters related to energy metabolism (cluster 1 including 142 genes), cell cycle (cluster 2, 175 genes), gene expression (cluster 3, 127 genes), anatomical structure development (clusters 4, 480 genes) and cell communication/immune response (cluster 5, 123 genes) were identified. For each cluster, some relevant GO BP terms and pathways (KEGG and WikiPathways) are presented in Table 3 and Table 4. Full details of enriched biological processes and pathways, enrichment score, adjusted P-value and number of gene present in each cluster are reported in the Table S4 and Table S5. Cluster 1 comprised 98 genes highly expressed in SM and 44 in LM. Significantly enriched GO BP terms (P-value <7.4E^−07^, enrichment score (ES): 1.4 to 15.2) and pathways (P-value <1.8E−02, ES: 3.2 to 38) were mainly related to energy metabolism. Cluster 1 genes were assigned to several enriched biochemical pathways including “Electron Transport Chain”, “Oxidative phosphorylation”, “Glycolysis and Gluconeogenesis”, “Fatty Acid Beta Oxidation” and “Citrate cycle (TCA cycle)”. The GO BP term “generation of precursor metabolites and energy” with the highest P-value, was associated with 39 genes encoding five mitochondrial electron transfer chain complex subunits that were mainly expressed in SM. Succinate dehydrogenase complex, subunit A, flavoprotein (Fp) (*SDHA*) and genes of long chain fatty acid metabolism (acyl-CoA dehydrogenase, very long chain, *ACADVL*) were also more expressed in SM whereas solute carrier family 25, member 27 (*SLC25A27* also known as uncoupling proteins 4 *UCP4*) and solute carrier family 25, member 14 (*SLC25A14* also known as uncoupling proteins *UCP5*), phosphorylase kinase, alpha1 and beta (*PHKA1*, *PHKB*) and phosphoenolpyruvate carboxykinase1 (*PCK1*) were overexpressed in LM. Cluster 2 included 73 and 102 highly expressed genes in SM and LM, respectively. Enriched GO BP terms (P-value <6.6E^−05^, ES: 1.1 to 7.5) and pathways (P-value <1.6E−02, ES: 2.7 to 7.6) were related to cell cycle process. “Cell cycle” and “Ubiquitin mediated proteolysis” were the most important enriched pathways associated with cluster 2. Genes overrepresented in SM were mainly linked to G1 phase: cyclin D2 and D3 (*CCND2*, *CCND3*). Genes overrepresented in LM were related to the control of cell cycle checkpoint, GO/G1, G1/S, S/G2 and G2/M transition and M phase: anaphase promoting complex subunit 1 and 4 (*ANAPC1*, *ANAPC4*), cell division cycle 26 and 27 (*CDC26*, *CDC27*) as well as DNA replication and DNA repair process. Cluster 3 contained 43 and 84 highly expressed genes in SM and LM, respectively. Cluster 3 enriched GO BP terms (P-value <1.6E^−07^, ES: 1.5 to 11.7) were related to gene expression. Significantly enriched pathways were related to “mRNA processing” (P-value = 1.5E−10, ES = 9.3) and “Spliceosome” (P-value = 1.7E−12, ES = 10.9). In this cluster, two of the four myogenic regulatory factors, myogenic differentiation 1 (*MYOD1*) and myogenic factor 6 (*MYF6* also known as *MRF4*) were overrepresented in SM. Cluster 4 was the biggest one with 288 and 192 genes overexpressed in SM and LM, respectively. Enriched GO BP terms were mainly related to anatomical structure development (P-value <1.7E^−05^, ES: 1.5 to 4.4). Among enriched pathways found associated with cluster 4, “ECM-receptor interaction” (P-value = 7.1E−07, ES = 5.9) and “Focal adhesion” (P-value = 1.1E−6, ES = 3.4) had the highest P-value. Genes overrepresented in LM were implicated in cell division, chromosomal organization, structural maintenance of sarcomere and sarcoplasmic protein: nebulin (*NEB*), titin (*TTN*), *RYR1* and triadin (*TRDN*). Genes overrepresented in SM were involved in cell migration, cell surface and extracellular matrix (ECM): cadherin 2, type 1, N-cadherin (*CDH2*), CD44 molecule (*CD44*), caveolin 3 (*CAV3*) and code for major constituent of the contractile apparatus: myosin, heavy chain 3, 8, 9 and 11 (*MYH3*, *MYH8*, *MYH9* and *MYH11*), troponin I type 3 (*TNNI3*) and troponin T type 2 (*TNNT2*). Differentially expressed genes that might control muscle size were either more expressed in LM, follistatin (*FST*) and myostatin (*MSTN*) or in SM, insulin-like growth factor 1 (somatomedin C) (*IGF1*), transforming growth factor, beta 1 and 3 (*TGFB1* and *TGFB3*). Last, cluster 5 contained 90 overexpressed genes in SM and 33 overexpressed genes in LM. Significantly enriched GO BP terms (P-value <2E−05, ES: 1.7 to 16.3) and biological pathways (P-value <1.E−12, enrichment score (ES): 2.6 to 39) were related to cell communication and inflammatory immune response. Interestingly, this cluster highlighted SM overexpressed genes also involved in the regulation of developmental and myogenesis signaling pathways or in the muscle regeneration process: fibroblast growth factor 18 (*FGF18*), members of the Notch signaling pathway (*NOTCH3* and delta-like 4, *DLL4*), chemokine (C-C motif) ligand 21 and 23 (*CCL21* and *CCL23*) and complement component and factor (complement component 1, q subcomponent, C chain, *C1QC*; complement component 1, r subcomponent, *C1R*; complement component 1, s subcomponent, *C1S*; complement component 3, *C3*; complement component 4A, *C4A* and complement factor B, *CFB*).

**Table 3:** Relevant biological processes significantly enriched in clustered differentially expressed genes.

Cluster^1^	ES^2^	Specific GO term^3^	nG^4^	P-value^5^
**1**		**Energy metabolism**	**142**	
	7.7	GO:0006091∼generation of precursor metabolites and energy	39	<1E^−12^
	4.7	GO:0006629∼lipid metabolic process	45	<1E^−12^
	5.1	GO:0055114∼oxidation reduction	41	<1E^−12^
	9.2	GO:0015980∼energy derivation by oxidation of organic compounds	24	<1E^−12^
	10.3	GO:0045333∼cellular respiration	18	<1E^−12^
**2**		**Cell cycle**	**175**	
	2.8	GO:0044267∼cellular protein metabolic process	110	<1E^−12^
	2.5	GO:0006464∼protein modification process	54	1.9E^−09^
	4.4	GO:0006281∼DNA repair	20	3.7E^−07^
	6.8	GO:0051439∼regulation of ubiquitin-protein ligase activity during mitotic cell cycle	9	4.3E^−05^
	2.3	GO:0007049∼cell cycle	32	4.7E^−05^
**3**		**Gene expression**	**127**	
	5	GO:0016070∼RNA metabolic process	113	<1E^−12^
	3.4	GO:0010467∼gene expression	119	<1E^−12^
	7.7	GO:0006396∼RNA processing	56	<1E^−12^
	10.2	GO:0008380∼RNA splicing	42	<1E^−12^
	3.1	GO:0045449∼regulation of transcription	66	<1E^−12^
	2.8	GO:0010468∼regulation of gene expression	68	<1E^−12^
**4**		**Anatomical structure development**	**480**	
	2.1	GO:0009653∼anatomical structure morphogenesis	87	3.4E^−10^
	2.5	GO:0007155∼cell adhesion	60	1.3E^−09^
	1.8	GO:0065008∼regulation of biological quality	97	3.4E^−08^
	3.2	GO:0003012∼muscle system process	27	1.6E^−06^
	2.8	GO:0007517∼muscle organ development	32	1.9E^−06^
**5**		**Cell communication/immune response**	**123**	
	3.1	GO:0007154∼cell communication	96	<1E^−12^
	3.2	GO:0007165∼signal transduction	89	<1E^−12^
	1.8	GO:0050794∼regulation of cellular process	97	<1E^−12^
	7.3	GO:0006955∼immune response	39	<1E^−12^
	6.2	GO:0006954∼ inflammatory response	17	1.9E^−08^

1 Differentially expressed genes were clustered using GO BP terms semantic similarity between genes as distance, to group functionally similar genes together.

2 Cluster enrichment score (ES).

3 Unique identifier and gene ontology term in the GO database (http://www.geneontology.org/).

4 n_G_, number of genes in the category.

5 Benjamini and Hochberg adjusted P value.

**Table 4:** Relevant biological pathways significantly enriched in clustered differentially expressed genes.

Cluster^1^	ES^2^	Pathway name	Pathways	nG^3^	P-value^4^
**1**		**Energy metabolism**		**142**	
	7.7	Metabolic pathways	KEGG	84	<1E^−12^
	15.7	Electron Transport Chain	Wikipathways	23	<1E^−12^
	12.9	Oxidative phosphorylation	KEGG	22	<1E^−12^
	13.1	Glycolysis and Gluconeogenesis	Wikipathways	8	1.7E^−07^
	7.5	Fatty Acid Beta Oxidation	Wikipathways	8	1.0E^−05^
	13.2	Citrate cycle (TCA cycle)	KEGG	5	4.6E^−05^
**2**		**Cell cycle**		**175**	
	6.6	Cell cycle	KEGG	11	9.8E^−06^
	5.3	Ubiquitin mediated proteolysis	KEGG	11	4.8E^−05^
	6.4	p53 signaling pathway	KEGG	6	1.0E^−03^
	5.1	Ribosome	KEGG	7	1.1E^−03^
	5.8	Gap junction	KEGG	6	1.2E^−03^
**3**		**Gene expression**		**127**	
	10.9	Spliceosome	KEGG	16	<1E^−12^
	9.3	mRNA processing	Wikipathways	15	1.5E^−10^
**4**		**Anatomical structure development**		**480**	
	5.9	ECM-receptor interaction	KEGG	15	7.1E^−07^
	3.4	Focal adhesion	KEGG	25	1.1E^−06^
	6.0	Striated Muscle Contraction	Wikipathways	12	7.6E^−06^
	2.9	Tight junction	KEGG	14	1.9E^−03^
	2.3	Regulation of actin cytoskeleton	KEGG	17	3.3E^−03^
	2.9	Cell adhesion molecules (CAMs)	KEGG	10	6.8E^−03^
**5**		**Cell communication/immune response**		**123**	
	13.8	Toll-like receptor signaling pathway	KEGG	11	2.0E^−09^
	39	Complement Activation, Classical Pathway	Wikipathways	5	1.9E^−07^
	5.0	Chemokine signaling pathway	KEGG	7	7.0E^−04^
	5.7	Cell adhesion molecules (CAMs)	KEGG	5	1.9E^−03^
	4.9	TNF alpha Signaling Pathway	Wikipathways	5	4.5E^−03^

1 Differentially expressed genes were clustered using GO BP terms semantic similarity between genes as distance, to group functionally similar genes together.

2 Cluster enrichment score (ES).

3 n_G_, number of genes in the category.

4 Benjamini and Hochberg adjusted P value.

## Discussion

Our objective was to clarify the biological events which could explain the muscle phenotypic differences reported in the literature between the LM and SM [3], [4], [14]. Since skeletal muscle is a heterogeneous tissue, transcriptome analysis of skeletal muscle may reflect mRNA composition of various cell types existing in this tissue. However, we assumed that myofibers are the main skeletal muscle component and that comparison between muscles is informative. The custom GenmascqChip [15] used in this study allows the analysis of 10753 probes and the identification of 5582 differentially expressed probes between LM and SM demonstrating that the GenmascqChip is a powerful tool to study pig muscle gene expression in order to gain a better understanding of muscle physiology. Furthermore, directions of differential gene expression FC observed in microarray analysis have been validated by quantitative PCR analyses using seven differentially expressed genes. We have considered that FC direction agreement between the two methods validates the differentially expressed genes set. Besides, there are few studies comparing gene expression of contrasted skeletal muscles in pigs, most of them focus on LM and none of them compared LM and SM [16], [17]. The number of genes found differentially expressed between these two muscles is surprisingly high. In fact, Hornshøj et al. [17] described a similar expression pattern between these two muscles, while the comparison of red and white skeletal muscles that are much more contrasted than LM and SM led to far fewer differentially expressed genes in pigs [16], [18] or mice [19]. However, different experimental conditions such as microarray platform technology, whole genome vs. focused microarray, sample size, samples pooling, FC threshold might account for this discrepancy. Several studies have compared gene expression level from different microarray technologies and relate divergence across the data generated [20]–[23]. Stretch et al. [24] have studied the effect of the sample size on differentially expressed gene discovery. They studied muscle gene expression on 134 samples (69 males, 65 females) and found that using sample of n = 10 (5 males, 5 females) results in no significant genes at P-values <0.0001, whereas larger sample size n = 120 (60 males, 60 females) identifies 472 differentially expressed genes at the same P-value cutoff. Anyway, this new finding reinforces the importance of gene expression variability between muscles which could affect muscle development and hence meat quality [13].

Microarray experiments result in list of hundred to thousand differentially expressed genes and the main objective of functional data analysis is to determine relevant biological interpretations. In this context, hierarchical clustering is often performed using gene expression correlation coefficient matrix as distance considering that co-expressed genes share the same biological processes. Biological knowledge is then used to identify enriched biological processes in each gene cluster [25]. Using this approach, we obtained two large clusters corresponding to over- and underrepresented genes which led to dozens of dissimilar enriched terms (data not shown). Furthermore, biological pathways are mostly controlled by the balance between up and downregulations. Performing functional analysis separately for up and downregulated genes list might result in loss of biological information since genes involved in the same pathway could have been assigned in different set. This partial information may leads to misinterpretation of differentially expressed gene list. Some pathways might then have been discarded because their enrichment value was deemed insufficient, whereas gene involved in the regulation of this pathways were present in up and downregulated genes list. To avoid this and create meaningful clusters, we used semantic similarity of GO BP terms to group functionally similar genes together. Wang’s metrics [26] was chosen over the information content-based semantic similarity measures because the latter require a reliable corpus in order to compute GO terms frequencies, and such a corpus does not exist for moderately studied species such as *Sus scrofa*. We successfully identified five functional clusters including both over and underrepresented genes. This approach was well suited to the size of our data set (around 1600 genes). However, the hierarchical clustering algorithm led to exclusive classification and we assume that clusters cannot overlap whereas genes may be involved in several biological processes. Thus most but not all relevant GO BP terms and pathways were highlighted with this procedure. The five main relevant biological networks associated to skeletal muscle differences were “energy metabolism”, “cell cycle”, “gene expression”, “anatomical structure development” and “cell communication/immune response”. Some examples of differentially expressed genes will be discussed in relation to energy metabolism and myogenic progenitor cells recruitment and sarcomerogenesis which composed steps of myogenesis process leading to the formation and growth of myofibers. The last part will discuss contrasted results in relation to muscle regeneration process.

### Energy Metabolism

Our functional analysis identified “energy metabolism” as one of the most relevant biological pathway associated to LM and SM differentially expressed genes set. SM overexpressed genes were related to mitochondrial fatty acid beta-oxidation pathway (*ACSF3*, *ACADVL*, *ACADS* and *HADHA*), citric acid cycle (*ACO2* and *SDHA*) and the five mitochondrial respiratory chain complex: NADH dehydrogenase (ubiquinone) subunits (*MT-ND3*, *MT-ND6*, *NDUFA3*, *NDUFA8*, *NDUFA9*, *NDUFA10*, *NDUFA11* and *NDUFV1*), succinate dehydrogenase subunits (*SDHA*), ubiquinol-cytochrome c reductase complex subunits (*CYC* and *UQCRC1*), cytochrome c oxidase subunits (*COX4I2*, *COX8A* and *MT-CO1*) and ATP synthase subunits (*ATP5A1* and *ATP5D*). On the other hand, LM overexpressed genes were related to glycogenolysis regulation (*PHKA1* and *PHKB*), pyruvate metabolism pathways (*PCK1*) and uncoupling protein (*UCP4* and *UCP5*). These results suggest on the one hand a higher mitochondrial oxidative activity in SM than in LM while on the other hand a limited usage of oxidative phosphorylation through uncoupling protein overexpression and a predominant usage of the anaerobic glycolytic pathway in LM. These results are consistent with and refine previous knowledge on SM and LM metabolic characteristics. In fact, these two glycolytic muscles are predominantly composed of fast-twitch type II fibers and low level of slow-twitch type I fibers. However, SM is composed of highest percentage of intermediate fast-twitch type IIa myofibers and exhibited higher oxidative capacity than LM [3], [4], [6].

### Myogenesis Process

Although mature myofibers are postmitotic cells, functional enrichment analysis highlighted cell cycle, gene expression and muscle development as important features to characterize contrasted LM and SM expression profiles. SM overexpressed genes were related to satellite cells activation (*IGF1*, *FGF18*), cell cycle control at G1 phase (*CCND2*, *CCND3* and cyclin-dependent kinase inhibitor, *CDKN1B*) and myoblast determination (*MYOD1*, *MRF4*). On the other hand, LM overexpressed genes were involved in the negative regulation of satellite cells activation (*MSTN*, *FST*), and cell cycle progression through G1/S, S/G2 or G2/M transition and in M phase. Hormonal control of satellite cells activation involved different growth factors including insulin-like growth factor I, which is a well-known hypertrophy factor acting on muscle mass and fibroblast growth factor [27], [28]. In fact, insulin-like growth factor I induced myogenesis by activating satellite cells and promoting proliferation, differentiation and fusion with existing myoblast [27]. On the other hand, myostatin and its antagonist follistatin, overexpressed in LM, are both involved in the main signaling pathway that negatively regulates satellite cells activation [29], [30]. *MSTN* is expressed in satellite cells and act on cell cycle progression to maintain the G1 resting state (G0) and limit muscle growth by inhibiting satellite cells activation and proliferation [31]. Follistatin antagonize myostatin inhibitory activity by direct protein interaction. Balance between follistatin and myostatin limit the recruitment of satellite cells [29].

Once activated, satellite cells proliferate before undergoing myogenic differentiation. Cells proliferation relies on kinases or E3 ubiquitin-protein ligases that regulate activity or stability (ubiquitination and subsequent proteasomal degradation) of key cell-cycle control proteins. Interestingly, we have identified “Cell cycle” and “Ubiquitin mediated proteolysis” among enriched pathways associated with cluster 2. Among E3 ubiquitin-protein ligases, Anaphase promoting complex/cyclosome (APC/C) genes (*ANAPC1*, *ANAPC4*, *CDC26*, *CDC27*) were overexpressed in LM. APC/C is a key regulator of the eukaryotic cell cycle acting on G0/G1 transition, through S and G2 phase, and during mitoses to ensure proper and correct succession of cell cycle key events [32]–[34]. On the other hand, Cyclin D (*CCND2*, *CCND3*) major regulatory proteins of the G1 phase and *CDKN1B* genes were overrepresented in SM. During G1 phase cyclin-dependent kinase inhibitor 1B binds to cyclin D-CDK4 complexes, and thus promote the cell cycle arrest at G1 [35].

Another step of myogenesis relies on myoblast determination and differentiation. Determination and differentiation of myoblast progenitor is governed by the expression of a family of four muscle specific transcription factors called myogenic regulatory factors (MRFs): *MYOD1*, *MYF6*, *MYF5* (myogenic factor 5) and *MYOG* (myogenin) [36]. *MYOD1* and *MYF6* were specifically overrepresented in SM. During satellite cells proliferation phase, *MYOD1* is expressed by activated satellite cells and governs myoblast lineage determination [36]–[38]. *MYF6* is expressed in undifferentiated proliferating cells as well as in differentiated myoblast. Myogenic factor 6 seems to be implicated in both roles: myogenic specification genes acting on activated satellite cells and terminal differentiation genes [27], [36], [37].

Collectively, this set of differentially expressed genes strongly suggests notable differences in myogenic progenitor recruitment, proliferation and differentiation between LM and SM. LM seems to limit satellite cells activation through myostatin pathway. However, once initiated, cell cycle seems to be completed in LM with overrepresentation of genes involved in the control of cell cycle key step (G1/S transition, S phase, S/G2 and G2/M transition, M phase). On the other hand, our results suggest that SM activates myogenic progenitors through insulin-like growth factor I, and that cells withdraw from cell cycle after mitosis at G1 phase allowing cell commitment to the differentiation program through MRFs expression.

Migration is a crucial step in myogenesis as it allows myoblasts alignment before fusion in myotubes. Myoblasts specifically fuse to each other to form myotube, and in a second phase fuse to existing myotubes for muscular development, muscular maintenance or regeneration process [39]. The largest functional cluster underlying skeletal muscle discrepancy was related to anatomical structure and muscle development GO BP terms and “ECM-receptor interaction” and “Focal adhesion” pathways. The extracellular matrix and cell adhesion molecule play an important role in myoblast mobility and fusion. *CDH2*, *CD44* and *CD164* (CD164 molecule, sialomucin), three genes coding for cell-surface glycoproteins, are involved in cell-cell interactions, cell adhesion and migration. CD44 and CD164 molecules are two transmembrane proteins playing a key role in myoblast motility regulation [40], [41]. Cadherins have been implicated in embryonic myoblast fusion, post-natal myogenesis or even regeneration process [42]–[44]. *CAV3* gene encodes an integral membrane protein, is induced during myoblast differentiation and has been implicated in myoblast fusion regulation and myotubes formation [45], [46]. Last, adiponectin (*ADIPOQ*) well known for its implication in glucose metabolic regulation, have been also implicated in an autocrine/paracrine signaling effects on myoblast differentiation and fusion [47], [48]. These genes are overrepresented in SM. Our results suggest that, in SM, myoblast migration, alignment and fusion in myotubes are more active than in LM. Moreover, genes encoding giant sarcomeric protein such as *NEB* and *TTN* and genes encoding proteins of the sarcoplasmic reticulum membrane calcium release channel such as *RYR1* and *TRDN* as well as genes encoding contractile proteins such *MYH3*, *MYH8*, *MYH9* and *MYH11*, *TNNI3* and *TNNT2* are differentially expressed between LM and SM. These genes are involved in terminal differentiation of myoblasts in sarcomerogenesis and in sarcomeric structure stabilization and maintenance [49]–[53]. This set of differentially expressed genes suggests that sarcomere assembly and maintenance processes are important process to characterize contrasted LM and SM expression profile.

Altogether, regarding satellite cells activation, myoblast differentiation and fusion to form sarcomere, our results suggest higher myogenic activity in SM than in LM.

### Muscle Regeneration Process

Last, functional enrichment analysis identified inflammatory immune response as relevant biological pathway to characterize LM and SM. SM overexpressed genes related to inflammatory response such as chemokine ligand (*CCL21* and *CCL23*) and complement component or factor (*C1QC*, *C1R*, *C1S*, *C3*, *C4A* and *CFB*). In accordance, several studies have reported an induction of replication factors and cyclins in early stage of proliferative phase following muscle injury. This induction is followed by upregulation of myogenic factor and cyclin dependant kinase inhibitors upon transition from proliferation phase to differentiation phase [54], as well as overexpression of genes involved in inflammatory process, myogenic differentiation and ECM remodeling [55]–[57]. Moreover, several genes products overexpressed in SM have been shown to be involved and specifically induced during muscle regeneration process. *ADIPOQ* is involved in the regenerative processes of skeletal muscle [47] whereas cadherins molecules are upregulated in activated satellite cells following injury [58]. Tenascin C and biglycan (*BGN*) two ECM glycoproteins are thought to be involved in muscle repair [59], [60]. *BGN* mRNA expression which is low in mature myofibers, is highly upregulated during muscle regeneration in myoblast and newly formed/regenerating myotubes and is concomitant with the expression of embryonic isoform of myosin by these new myotubes [61]. Interestingly, SM re-expressed the embryonic (*MYH3*) and peri-natal (*MYH8*) isoforms of myosin heavy chain which could be associated with muscle regeneration. In fact, embryonic and peri-natal myosin isoforms disappear at birth and are progressively replaced by adult MHC [62], [63] but re-expression of these developmental myosin isoform has already been reported during muscle regeneration [27], [64]. Finally, insulin-like growth factor I have been implicated in muscle regeneration process and acting through a paracrine/autocrine regulation [27]. Thus, SM expression profile strongly suggests a regenerative muscular process which is characterized by expression of genes related to inflammatory response, fetal myogenic program and ECM proteins. While SM is used for locomotion, it could be therefore physiologically more active and subject to more minor lesions than the LM which is required for postural purpose. Thus, in SM, satellite cells might be activated and process to proliferation, myoblast differentiation and fusion for either muscle homeostasis or to form new multinucleated myotubes [27], [65]–[67]. Concomitantly, the mechanism of sarcomere maintenance has to incorporate newly synthesized contractile proteins [68].

In conclusion, our study aimed to identify the biological events that underlie the differences between LM and SM metabolic and contractile properties by comparing their gene expression profiles. Results shed light on differentially expressed genes mainly related to myogenesis processes which suggests dissimilar post-natal myogenic activity between the two muscles. However we cannot presume if this results from dissimilar muscle maturity and/or from regeneration process occurring mainly in SM. This variability could affect muscle development and hence meat quality traits [13], [69], thus skeletal muscle specificity should be taken into account to determine important features for meat quality traits and identify useful biomarkers of pork quality.

## Methods

### Ethics Statement

All samples analyzed in this study were collected post-mortem, from pigs raised and slaughtered in the context of pig meat production. These animals and the scientific investigations described herein are therefore not to be considered as experimental animals per se, as defined in EU directive 2010/63 and subsequent national application texts. Consequently, we did not seek ethical review and approval of this study as regarding the use of experimental animals. All animals were reared and slaughtered in compliance with national regulations pertaining to livestock production and according to procedures approved by the French Veterinary Services. Pigs were raised on the France Hybrides nucleus herd of Sichamps and slaughtered on the ORLEANS Viandes commercial EU approved slaughterhouse according to standard procedures (ORLEANS Viandes, Fleury-les-Aubrais, France).

### Animals and Study Design

Analyses presented here were performed on a subset of larger cohort, and defined as two half sibs family of 41 and 49 animals. The 90 pigs used in this study were non modified domestic pigs produced as an intercross on two successive generations between two terminal sire lines (FH016, Pietrain type line, and FH019 synthetic line from Duroc, Large White and Hampshire founders, FRANCE HYBRIDES, St Jean de Braye, France). See Cherel et al. [70] for details. All animals were raised on the same farm and slaughtered at an average body weight of 108 kg in the same commercial slaughterhouse according to standard procedures for commercial slaughtering (ORLEANS Viandes, Fleury-les-Aubrais, France). Average age was 151 days at slaughtering. Slaughtering method entailed exsanguination following electric stunning. The Longissimus and Semimembranosus muscles were sampled 20 minutes post-mortem at the same time point following exsanguination from the same carcasses. To minimize biopsy site variation, each sample type (i.e. LM or SM) was collected by a single operator. Muscle samples were collected using a manual trocar instrument and are localize in the superficial regions of the muscle. Muscle sample were immediately frozen in liquid nitrogen. All animals were genotyped as homozygous wild type genotypes NN and rn+rn+ with regard to the HAL and RN loci, respectively [71], [72].

### Total RNA Extraction

Total RNA was extracted by crushing the frozen tissue in Trizol reagent (Invitrogen, Cergy-Pontoise, France) and purified using Nucleospin RNA II Kit (Macherey-Nagel, Lyon, France). Total RNA was quantified using a NanoDrop ND-1000 spectrophotometer (Thermo Scientific, Illkirch, France) and the integrity was assessed using the Agilent RNA 6000 Nano kit with an Agilent 2100 Bioanalyzer (Agilent Technologies France, Massy, France). The RNA integrity number (RIN) was above eight for all samples.

### Microarray Design

The “GenmascqChip”, a custom 15 k pig skeletal muscle microarray was used in this study [15]. Microarray annotation was produces using BLAST 2.2.23+ [73] for megaBLAST analysis of the 15198 oligonucleotides sequences (60 mers) printed on the microarray against ENSEMBL cDNA and NCBI refSeq mammal databases. Annotation was based on similarity and quality criteria [74]. Among the 15198 probes of the GenmascqChip, 12939 probes (i.e. 85% of the oligonucleotides) have been linked to a unique annotated sequence and to 9169 unique genes (i.e., 30% of redundancy). An 8×15 K oligo-microarray Agilent format was chosen, therefore one probe per microarray and eight microarrays were fitted in each slide. Description of the GenmascqChip is publicly available into the GEO repository (http://www.ncbi.nlm.nih.gov/geo/) through GEO platform accession no. GPL11016.

### Microarray Hybridization

Total RNA (350 ng) from each sample (90 LM, 90 SM) was individually labeled with Cy3 using the Low RNA Input Linear Amplification Kit PLUS, One-Color (ref 5188–5339, Agilent Technologies, Massy, France) following the manufacturer’s instructions. Microarray hybridizations were carried out at 65°C for 17 hours in Agilent’s SureHyb Hybridization Chambers containing 600 ng of Cy3-labeled cRNA sample. Slides were disassembled and washed in Gene Expression Wash Buffer 1 for 1 minute at room temperature and then in Gene Expression Wash Buffer 2 for 1 minute at 37°C. Microarrays were scanned at 5 µm/pixel resolution using the Agilent DNA Microarray Scanner G2505B, and images were analyzed with Agilent Feature Extraction Software (Version 9.5), using the GE2-v5_95_Feb07 FE extraction protocol. These MIAME compliant microarray data have been deposited into the GEO repository (http://www.ncbi.nlm.nih.gov/geo/) and are publicly available through GEO Series accession no. GSE33957.

### Microarray Data Analysis

All statistical analyses were performed using R software version 2.8.1 [75]. Raw spots intensities were first submitted to quality filtration based on intensity, uniformity and saturation criteria. All probes with more than 50% quality flagged spots within muscle were deleted from this study whereas remaining probes were considered significantly expressed in skeletal muscle. Processed signal intensities from filtered probes were natural log transformed and centered within sample by subtraction of the sample median value. Within probes, all spots that deviated by more than three times the standard deviation from the mean were considered as outliers and deleted from further analysis. All probes whose spots were flagged or detected as outliers within more than 50% of samples per muscles were also removed from the analysis. To increase the robustness of differential expression analysis, probes with the smallest expression variability across samples were filtered out using K-means algorithm (k = 3) [76]. For each remaining probes, raw expression data were analyzed according to the following linear model of variance:

Y = Sex+Slaughter Batch+Hybridization Batch within Sire+Carcass Weight+Muscle+E.

Where Y is the raw expression data; Sex is the fixed effect of sex (2 levels), Slaughter Batch (2 levels) represent the effect of the slaughter season (summer or winter); Hybridization Batch within Sire represent the effect of the hybridization batch per Sire family (9 levels for Sire Family level 1, 11 levels for Sire Family level 2); Carcass Weight is a covariable to take into account the animal weight at slaughter time; Muscle is the fixed effect of the Muscle (2 levels) and E is the residual. All effects exceeding the significant level of P<0.2, were kept in the model. The muscle effect was kept in each model. P-values were adjusted according to Benjamini and Hochberg multiple testing correction procedure [77]. Differentially expressed probes were selected using adjusted P-values of 0.05 or less. Adjusted means for LM and SM were calculated using lsmeans function for the muscle effect (package lsmeans). Differentially expressed probes were assigned as overrepresented in LM or overrepresented in SM according to the greatest mean. Fold change value is expressed as ratio of the greatest to the least mean: the expression ratio of LM to SM muscle effect when genes are overrepresented in LM and as the expression ratio of SM to LM muscle effect when genes are overrepresented in SM.

### Validation of Microarray Data: Reverse Transcription and Quantitative Real Time PCR (RT-qPCR)

RT-qPCR was performed using SYBR Green methodology to validate seven differentially expressed genes: ADAM metallopeptidase with thrombospondin type 1 motif 8 (*ADAMTS8*); aldolase A fructose-bisphosphate (*ALDOA*); CCAAT/enhancer binding protein alpha (*CEBPA*); muscle carnitine palmitoyltransferase 1B (*CPT1B*); diacylglycerol O-acyltransferase 2 (*DGAT2*); ryanodine receptor 1 (*RYR1*) and transforming growth factor beta 1 (*TGFB1*). Eight animals identified as non-outliers in microarray analysis were randomly chosen to validate those genes. Complementary DNA was synthesized from 2 µg of total RNA previously used in microarray analysis, using High Capacity cDNA Reverse Transcription Kit (Applied Biosystems, Foster City, CA). Primers (Table 5) were designed from porcine sequences using Primer Express software 3.0 (Applied Biosystems). Amplification was performed in triplicate, in 12.5 µl with 5 ng of reverse-transcribed RNA and both forward and reverse primers (200 nM each) in 1X PCR buffer (Fast SYBR Green Master Mix, Applied Biosystems) containing Uracil DNA glycosylase to prevent any DNA contamination from previous PCR. A StepOnePlus™ Real Time PCR system (Applied Biosystems) was used. Thermal cycling conditions were as follows: 50°C for 2 min, 95°C for 20 s, followed by 40 cycles of denaturation at 95°C for 3 s and annealing at 60°C for 30 s. Specificity of the amplification products was checked by dissociation curves analysis. Three genes were selected as stable reference genes for normalization using geNorm algorithm [78]: beta 2 microglobulin (*B2M*), TATAbox binding protein (*TBP*) and *18S* (*18S* rRNA predeveloped TaqMan kit from Applied Biosystems). For each sample, a normalization factor (NF) was calculated using geNorm algorithm and used for subsequent normalization. The normalized expression level (N_exp_) was calculated according to the following formula: N_exp_ = E^−ΔCt (sample-calibrator)^/NF, where the calibrator is a pool of the 16 skeletal muscle samples and E is the PCR efficiency calculated from the slope of calibration curve. Normalized expression levels of mRNAs were then compared between muscle using the Student t-test and P-value ≤0.05 for significance.

**Table 5:** Primers pairs used in quantitative real-time RT-PCR.

Target gene^1^	GenBank Accession^2^	Primers pair sequences (Forward/Reverse)
**ADAMTS8**	BI360058	ACCCCTCCAGCTATGGCTACA
		TGGATGGCTCCGCTGTTT
**ALDOA**	CB286787	CGCTGTCCCTGGGATCAC
		GCACTTGTTGATGGCGTTGA
**CEBPA**	AF103944.1	GTGGACAAGAACAGCAACGA
		CTCCAGCACCTTCTGTTGAG
**CPT1B**	AF284832	CACTGTCTGGGCAAACCAAA
		GCCACCTGGTAGGAACTCTCAAT
**DGAT2**	DT325702	CCTGATGTCTGGAGGCATCTG
		CACGATGATGATGGCATTGC
**RYR1**	M91451	CCCTGTGTGTGTGCAATGG
		GTTTGTCTGCAGCAGAAGCT
**TGFB1**	AF461808	AGCGGCAACCAAATCTATGATAA
		CGACGTGTTGAACAGCATATATAAGC
**B2M** 3	DQ178123	AAACGGAAAGCCAAATTACC
		ATCCACAGCGTTAGGAGTGA
**TBP** 3	DQ845178	AACAGTTCAGTAGTTATGAGCCAG
		AGATGTTCTCAAACGCTTCG

1 ADAMTS8, ADAM metallopeptidase with thrombospondin type 1 motif, 8; ALDOA, aldolase A, fructose-bisphosphate; B2M, beta-2-microglobulin; CEBPA, CCAAT/enhancer binding protein (C/EBP), alpha; CPT1B, carnitine palmitoyltransferase 1B (muscle); DGAT2, diacylglycerol O-acyltransferase 2; RYR1, ryanodine receptor 1 (skeletal); TBP1, TATA box binding protein; TGFB1, transforming growth factor, beta 1.

2 Accession number of the Sus scrofa sequence used to design primers.

3 Gene used as reference for normalization.

### Functional Analysis

To facilitate functional categorization of differentially expressed genes, we used hierarchical clustering of genes based on semantic similarity of GO BP terms. Semantic similarity was calculated between each pairwise combination of differentially expressed genes with a muscle fold change ratio (i.e. ratio of the greatest to the least muscle effect) above 1.5. Similarity was computed according to semantic similarity measures based on the method of Wang [26] implemented in the GOSemSim package [79]. Two genes that shared several GO BP terms result in similarity value close to one, indicating that they are similar in terms of biological process. Conversely dissimilar genes result in similarity value close to zero. Then hierarchical clustering was performed using “1-semantic similarity” as distance between two genes (similar genes have a distance close to zero) and “ward” as aggregation criterion to identify clusters of genes that share BP terms.

Functional characterization of clustered genes was performed using Gene Set Analysis Toolkit V2 (WebGestalt, http://bioinfo.vanderbilt.edu/webgestalt/) [80], [81] using BP GO terms, KEGG pathways [82] and WikiPathways [83]. The lists of genes were uploaded using orthologous human ENTREZ gene ID. A minimum of five genes was required for a term to be considered of interest. For each terms of interest, significance levels were calculated following a hypergeometrical test using GenmascqChip 15K orthologous human ENTREZ gene ID as background. A multiple testing correction P-value was calculated according to Benjamini and Hochberg procedure and an adjusted P-value of 0.05 or less was retained for significance.

## Supporting Information

Table S1 Genes overexpressed in *Longissimus*. Results were expressed as the *Longissimus* to *Semimembranosus* ratio of the gene expression. The P-value of each gene was adjusted according to the Benjamini and Hochberg procedure. Differentially expressed probes were selected using adjusted P-values of 0.05 or less. Redundancy represented the number of probes per gene. In this list, 240 genes had more than one probe.
(XLSX)

Table S2 Genes overexpressed in *Semimembranosus*. Results were expressed as the *Longissimus* to *Semimembranosus* ratio of the gene expression. The P-value of each gene was adjusted according to the Benjamini and Hochberg procedure. Differentially expressed probes were selected using adjusted P-values of 0.05 or less. Redundancy represented the number of probes per gene. In this list, 413 genes had more than one probe.
(XLSX)

Table S3 Functional cluster composition. Annotated differentially expressed genes with a muscle fold change above 1.5 were clustered based on their functional annotation (GO BP) semantic similarity. Hierarchical clustering was performed using “1-semantic similarity” as distance between two genes (similar genes have a distance close to zero) to identify clusters of genes sharing BP terms.
(XLSX)

Table S4 Enriched biological process of clustered differentially expressed genes. Functional characterization of clustered genes was performed using Gene Set Analysis Toolkit V2 (WebGestalt, http://bioinfo.vanderbilt.edu/webgestalt/) using BP GO terms. The lists of genes were uploaded using orthologous human ENTREZ gene ID. A minimum of five genes was required for a term to be considered of interest. For each terms of interest, significance levels were calculated following a hypergeometrical test using GenmascqChip 15 K orthologous human ENTREZ gene ID as background. A multiple testing correction P-value was calculated according to Benjamini and Hochberg procedure and an adjusted P-value of 0.05 or less was retained for significance.
(XLSX)

Table S5 Enriched pathways of clustered differentially expressed genes. Pathway analysis of clustered genes was performed using Gene Set Analysis Toolkit V2 (WebGestalt, http://bioinfo.vanderbilt.edu/webgestalt/) using KEGG pathways and WikiPathways. The lists of genes were uploaded using orthologous human ENTREZ gene ID. A minimum of five genes was required for a term to be considered of interest. For each terms of interest, significance levels were calculated following a hypergeometrical test using GenmascqChip 15 K orthologous human ENTREZ gene ID as background. A multiple testing correction P-value was calculated according to Benjamini and Hochberg procedure and an adjusted P-value of 0.05 or less was retained for significance.
(XLSX)
