# Correction: The *Longissimus* and *Semimembranosus* Muscles Display Marked Differences in Their Gene Expression Profiles in Pig

**DOI:** 10.1371/journal.pone.0100113

**Published:** 2014-06-06

**Authors:** The PLOS ONE Staff

The images for Figure 2 and Figure 3 are switched. Please see the correct figures and legends here.

**Figure 2:**
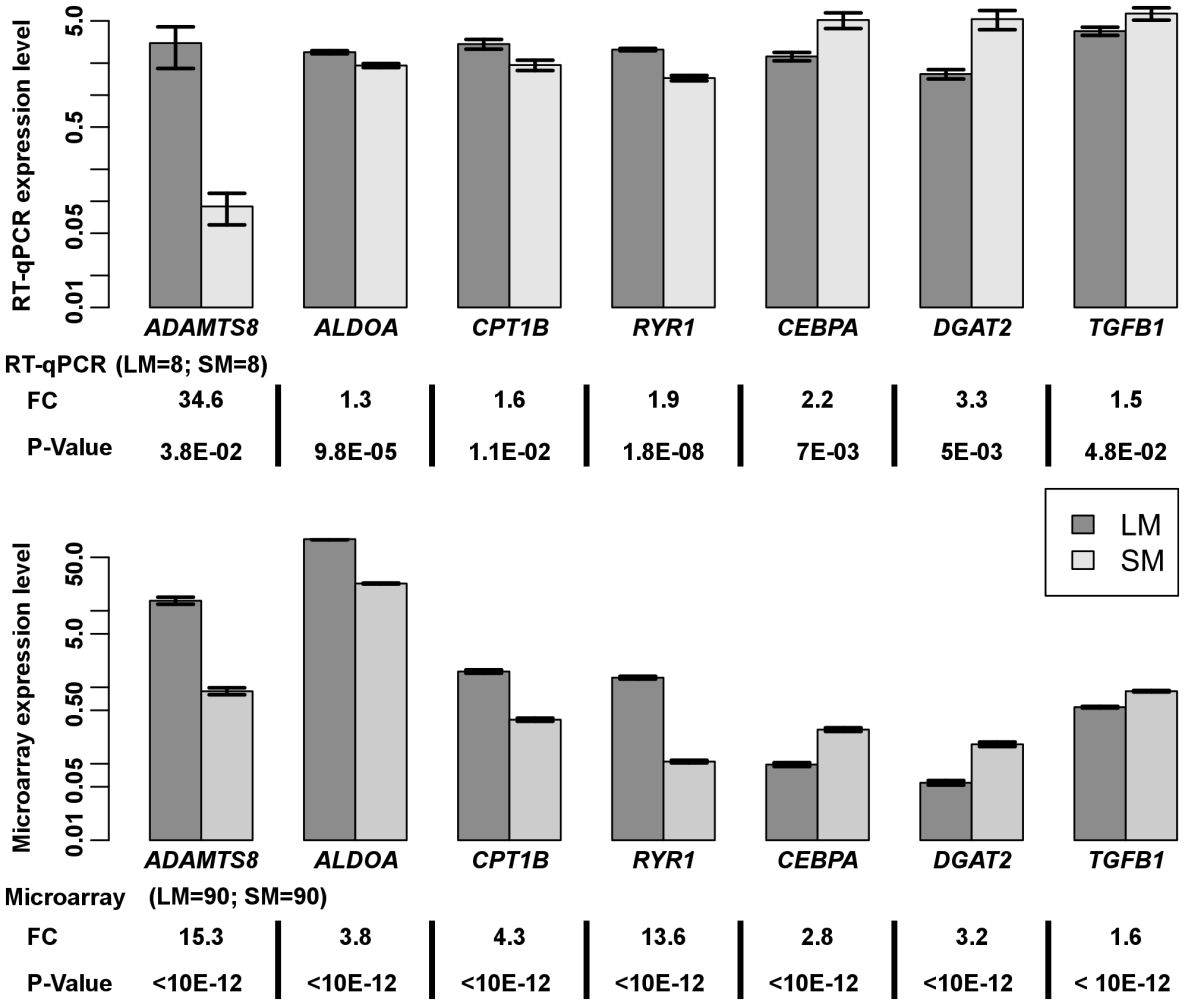
Validation of seven microarray differentially expressed genes between Longissimus (LM) and Semimembranosus (SM) muscles by quantitative RT-PCR. mRNA level is expressed using arbitrary units. Quantitative RT-PCR expression levels (LM = 8, SM = 8) were normalized to the expression of beta 2 microglobulin (*B2M*), TATAbox binding protein (*TBP*) and *18S* using geNorm algorithm. Microarray adjusted means for LM and SM (LM = 90; SM = 90) were calculated using least square means for the muscle effect. Data are expressed as means±s.d. Statistical significances are reported below the plot as Benjamini and Hochberg adjusted P-value for microarray data and as Student t-test P value for q RT-PCR. Fold change ratio is expressed as the expression ratio of LM to SM when genes are overrepresented in LM and as the expression ratio of SM to LM when genes are overrepresented in SM.

**Figure 3:**
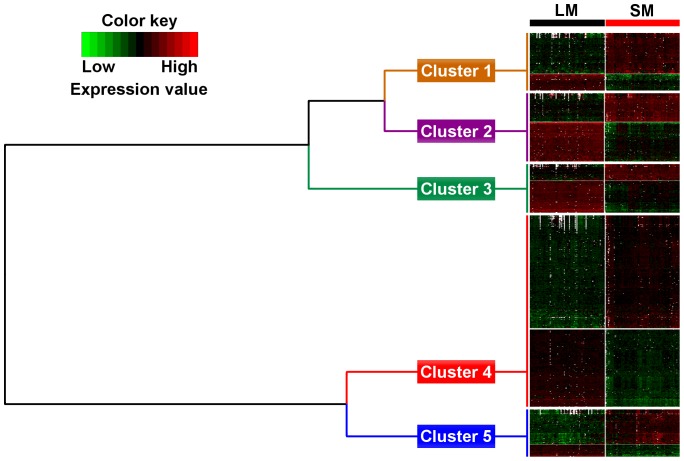
Hierarchical clustering of differentially expressed genes according to their GO BP terms semantic similarity. Annotated differentially expressed genes with a muscle fold change above 1.5 were clustered based on their functional annotation (GO BP) semantic similarity. Hierarchical clustering was performed using “1-semantic similarity” as distance between two genes (similar genes have a distance close to zero) to identify clusters of genes sharing BP terms. Five clusters were identified. Cluster 1 comprised 98 genes highly expressed in SM and 44 in LM. Cluster 2 included 73 highly expressed genes in SM and 102 in LM. Cluster 3 contained 43 highly expressed genes in SM and 84 in LM. Cluster 4 comprised 288 genes overexpressed in SM and 192 in LM. Cluster 5 involved 90 overexpressed genes in SM and 33 overexpressed genes in LM.
